# Cancer Treatment Using Nanofibers: A Review

**DOI:** 10.3390/nano14151305

**Published:** 2024-08-02

**Authors:** Muhammad Qamar Khan, Muhammad Abbas Alvi, Hafiza Hifza Nawaz, Muhammad Umar

**Affiliations:** 1 Department of Textile Engineering, School of Engineering and Technology, National Textile University, Faisalabad 37610, Pakistan; 2 Department of Materials, The University of Manchester, Manchester M13 9PL, UK; hafizahifza.nawaz@manchester.ac.uk

**Keywords:** cancer treatment, coaxial electrospinning, nanofiber, controlled drug delivery, Biocompatible polymer

## Abstract

Currently, the number of patients with cancer is expanding consistently because of a low quality of life. For this reason, the therapies used to treat cancer have received a lot of consideration from specialists. Numerous anticancer medications have been utilized to treat patients with cancer. However, the immediate utilization of anticancer medicines leads to unpleasant side effects for patients and there are many restrictions to applying these treatments. A number of polymers like cellulose, chitosan, Polyvinyl Alcohol (PVA), Polyacrylonitrile (PAN), peptides and Poly (hydroxy alkanoate) have good properties for the treatment of cancer, but the nanofibers-based target and controlled drug delivery system produced by the co-axial electrospinning technique have extraordinary properties like favorable mechanical characteristics, an excellent release profile, a high surface area, and a high sponginess and are harmless, bio-renewable, biofriendly, highly degradable, and can be produced very conveniently on an industrial scale. Thus, nanofibers produced through coaxial electrospinning can be designed to target specific cancer cells or tissues. By modifying the composition and properties of the nanofibers, researchers can control the release kinetics of the therapeutic agent and enhance its accumulation at the tumor site while minimizing systemic toxicity. The core–shell structure of coaxial electrospun nanofibers allows for a controlled and sustained release of therapeutic agents over time. This controlled release profile can improve the efficacy of cancer treatment by maintaining therapeutic drug concentrations within the tumor microenvironment for an extended period.

## 1. Introduction

Cancer is the second leading cause of death in the world, surpassed only by heart diseases. In the United States alone, it results in approximately 1620 deaths daily, highlighting its significant impact on public health. This statistic underscores the urgent need for continued research, enhanced prevention strategies, and the development of more effective treatments. Cancer has emerged as a formidable global health challenge, with its burden escalating over the years. The incidence and mortality rates associated with this disease have shown a steady upward trend. For instance, the global number of new cancer cases surged from approximately 14 million in 2010 to 19.3 million in 2020, reflecting a substantial increase of 37.8%. Similarly, cancer-related deaths climbed from 8.2 million in 2010 to 9.9 million in 2020, underscoring the disease’s devastating impact. These figures not only highlight the growing prevalence of cancer but also emphasize the urgent need for comprehensive prevention, early detection, and effective treatment strategies to combat this global health crisis [1].

**Figure 1:**
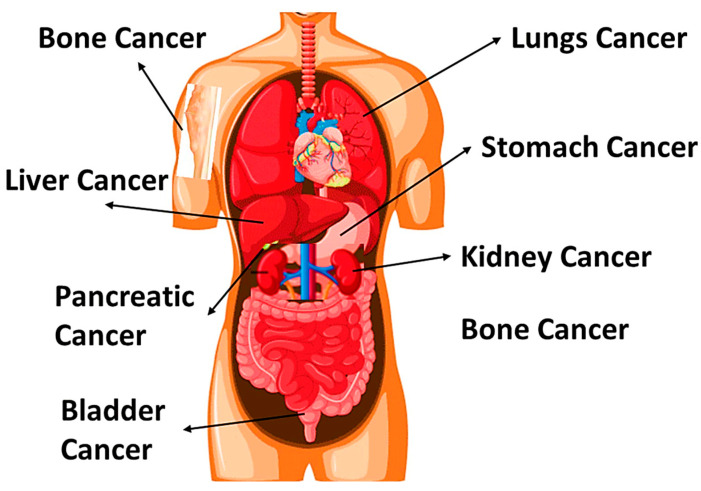
Types of cancer.

Cancer encompasses a diverse group of diseases, with prevalent types receiving global attention including but not limited to brain, breast, kidney, melanoma, and liver cancers as mentioned in Figure 1. Each type of cancer originates in specific tissues and exhibits unique characteristics, affecting various organs. Understanding these cancer types is crucial for effective diagnosis, treatment, and research efforts to improve outcomes and reduce the global burden of cancer. A comprehensive awareness of and research in these areas contribute to advancements in oncology, fostering better strategies for prevention, early detection, and personalized therapies [2].

The higher death rates for cancer observed in high-income countries can be linked to advanced healthcare infrastructures that enable more thorough diagnosis and reporting, alongside factors like increased life expectancy, and lifestyle factors like diet and tobacco use. Cancer is a complex disease influenced by a multitude of factors. Environmental factors play a significant role, with tobacco smoke, radiation, pollution, and occupational hazards being primary culprits. Exposure to these elements can increase the risk of developing various cancer types. Genetic factors also contribute, as inherited mutations can predispose individuals to certain cancers. While not all cancers are hereditary, family history remains a crucial consideration. Lifestyle factors significantly impact cancer risk, with diet, physical activity, obesity, alcohol consumption, and hormone replacement therapy being key influences. A diet rich in fruits and vegetables combined with regular exercise can help mitigate the risk, while unhealthy habits can increase it. Other factors such as age, immune system function, and infectious agents also play a part in cancer development. It is essential to note that the interplay of these factors varies across different cancer types, and while genetics can influence susceptibility, lifestyle modifications and environmental protections remain crucial in cancer prevention. In contrast, lower-income countries often face challenges in accessing quality healthcare, resulting in underdiagnosis and underreporting, contributing to apparent lower cancer death rates [3].

Nanofiber-based drug delivery systems offer a promising approach for achieving controlled and sustained drug release. However, the successful translation of these systems from bench to bedside necessitates a comprehensive understanding of their degradation profile and pharmacokinetic/pharmacodynamic (PK/PD) behavior. The rate and mechanism of nanofiber degradation significantly influence drug release kinetics, with biodegradable polymers offering the advantages of controlled release and the eventual elimination of the carrier system. Concurrently, elucidating the absorption, distribution, metabolism, and excretion of the encapsulated drug is crucial for optimizing dosage regimens and predicting therapeutic outcomes. By meticulously characterizing these parameters, researchers can design nanofiber-based systems that effectively deliver therapeutic agents while minimizing adverse effects.

Metastatic cancer poses a significant threat as it can spread from its original site to other parts of the body, forming secondary tumors. The majority of cancer-related mortality is often attributed to metastasis, as it makes treatment more challenging and increases the complexity of managing the disease. Effective strategies for early detection and intervention are crucial in addressing metastatic cancer and improving patient outcomes [4].

## 2. Current Cancer Treatments and Their Limitations

**Figure 2:**
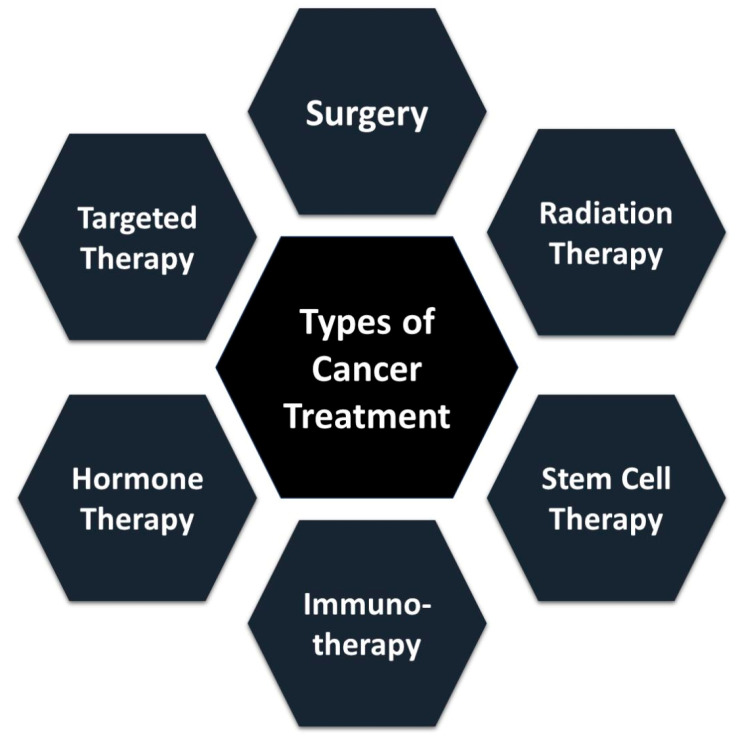
Types of cancer treatments.

For effective treatment of malignant tissues, comprehensive knowledge of the affected tissue, its condition, and treatment methodologies is essential. Various techniques, including hyperthermia/ultrasound, hyperthermia/chemotherapy, hyperthermia/radiotherapy, and hyperthermia/microwave, are utilized in cancer treatment (Figure 2) [5]. However, these machine-driven strategies may induce toxicity in specific tissues [5].

To date, seventy types of conventional anticancer drugs have been utilized as a therapy for cancer affected people. The utilization of these synthetic anticancer drugs can lead to harmful effects like hair loss, blood disorders, and nervous system issues [6,7]. While functionalized magnetic nanoparticles hold significant promise in biomedical applications, it is essential to acknowledge their limitations. The inability of these nanoparticles to traverse the blood–brain barrier poses a considerable challenge for targeting central nervous system diseases. Additionally, concerns regarding potential liver toxicity due to nanoparticle accumulation cannot be overlooked. Rigorous safety assessments and careful dose selection are crucial in mitigating these risks. To fully harness the potential of these nanoparticles, future research should prioritize developing strategies to enhance biodistribution and address safety concerns.

Several products extracted from plants like vincristine [8], vinblastine [9,10], paclitaxel [11,12], docetaxel [13,14], topotecan [15,16], irinotecan [17,18], flavopiridol, acronyciline, bruceantin, and thalicarpin [19,20,21,22] have been explored as natural anticancer agents. These anticancer agents are useful but also have numerous limitations such as low solubility, poisonousness, a shorter halftime, the dynamic release of anti-cancer drugs, and to the possibility of harming healthy cells [23].

The effective targeting of anticancer agents specifically to diseased tissues necessitates their navigation through various physiological barriers, including cellular membranes, extracellular spaces, bloodstreams, and specific organs. The unintended effects of these agents on healthy tissues due to non-specific distribution can lead to adverse side effects and escalate the cost of cancer treatment. This underscores the need for precision in drug delivery to maximize therapeutic efficacy while minimizing harm and financial burden.

Anticancer drug delivery systems have drawn attention due to their ability to (1) adjust the quantity of drug loaded [24], (2) enable precise, localized release of drugs, reducing cytotoxic effects on healthy tissues [25], (3) incorporate multiple therapeutic agents [26], (4) prevent premature burst release in targeted tissues [25], (5) ensure consistent release of drug payloads in targeted areas [27], and (6) offer biodegradable properties, enhancing safety and effectiveness.

Several considerations are crucial when selecting drug delivery carriers, including their structure, surface characteristics, and chemical composition. Currently, a diverse range of carriers are used for drug delivery, such as solid lipid nanoparticles, liposomes [28,29,30], compounds of silicate [31], compounds of magnetite [32,33], compounds of natural and synthetic polymers [34], quantum dots [35], carbon compounds [36,37], and metallic nanoparticles [38,39]. Additionally, various forms like patches [40], intravaginal rings [41], fibers [42], film [43], tablets [44], hydrogels [45], cervical caps [46], and sponges [47] are employed. While some materials primarily serve as carriers, others offer additional benefits in cancer treatment, underscoring the importance of selecting the appropriate carrier based on specific therapeutic needs [31,48,49].

**Figure 3:**
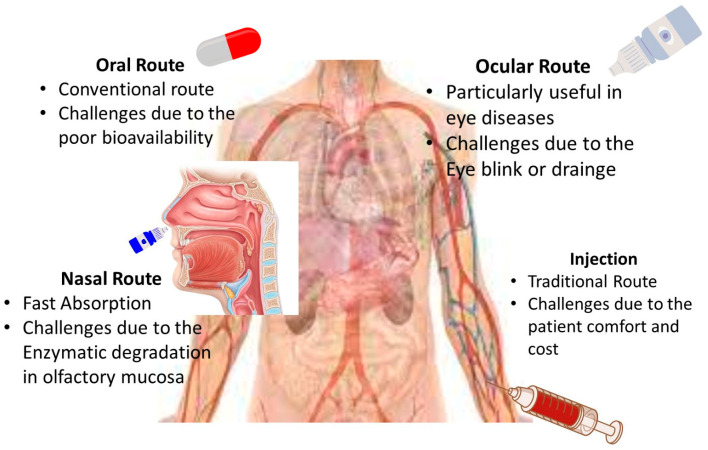
Drug delivery in human beings.

The various types of carriers demonstrate different degrees of effectiveness in drug delivery as mentioned in Figure 3. However, for both natural and biomedical applications, carriers must exhibit essential characteristics: (1) the manufacturing process should be straightforward and cost-effective; (2) the materials used for the carrier’s synthesis, including solvents and precursors, must be non-toxic and affordably priced; and (3) carriers must be biocompatible, renewable, and biodegradable, aligning with safety and environmental standards.

## 3. Importance of Nanofibers in Cancer Treatment

**Figure 4:**
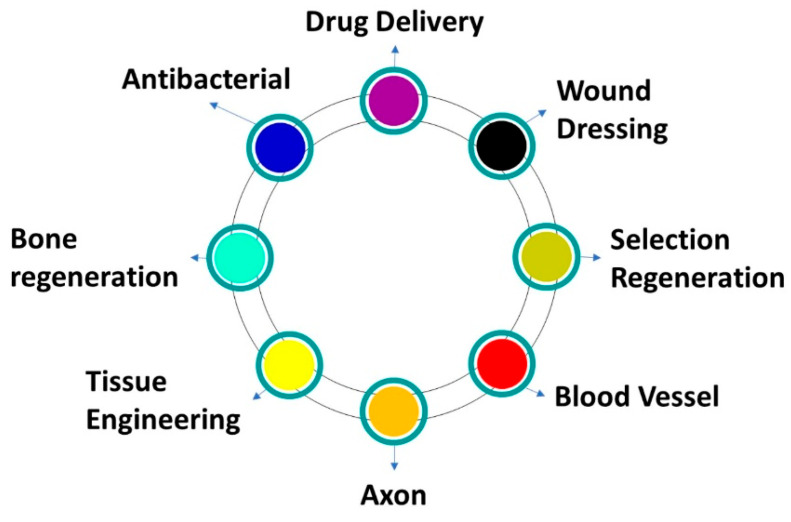
Applications of nanofibers in the medical field.

Nanomaterials have received much more attention as potential carriers in clinical and biomedical applications [50,51,52,53,54,55,56,57,58,59] due to their outstanding properties like the convenient processes used for their production at large scales, their enhanced mechanical characteristics, extremely permeable structure, high surface to volume ratio, analog to extra cellular model, and adjustable combination procedure [60,61,62]. Nanofibers are versatile materials with a broad spectrum of applications in the biomedical field. As illustrated in Figure 4, they excel in drug delivery, tissue engineering, and wound healing. Moreover, nanofibers’ high surface area makes them ideal candidates for biosensor development. By immobilizing biomolecules on their surfaces, these biosensors can detect cancer biomarkers with enhanced sensitivity, facilitating early-stage cancer diagnosis. Nanofibers are also efficiently used for medical applications as mentioned in the Figure 4 below. 

These applications include bone tissue engineering [63], medicinal compositions [64], scaffolds and artificial organs [65], wound dressing [50,66,67,68], drug delivery and medical engineering [69], antifungal [70] and anticancer [71] agents, antibacterial dressings [72], antibiotics [66], antimicrobials [73], medical prostheses [74], and postoperative implants [75,76].

Compounds of one dimension with thicknesses of 50 to 500 nm and length–width proportions of more than 1:20 that are composed of melts or polymer solutions are called nanofibers. Nanofibers [77,78,79,80,81,82,83,84,85,86] have permeable walls, are multi-layer and have ribbon, multi-channel, single layer constructions, necklace-like and weblike structures and core–shell structures [87,88,89,90,91,92]. The composition and breadth of nanofibers can be changed by changing numerous parameters like the polymer solution, viscosity, and method of preparation. Nanomaterials are the only material that can encapsulate more of a drug due to their specific size and structure [93]. Nonetheless, by using nanofibers with a single layer as medication transporters, the primary burst deliverance of medications may occur when a treatment is started [94]. On the other hand, the core shell and multi-layer nanofibers never show the phenomenon of self-releasing drugs [95,96,97] because, in core–shell nanofibers and multi-layer nanofibers, drugs are packed in the internal core or layers of fibers [98,99].

Nanofibers are also a cause of delay in drug delivery or release in core–shell nanofibers or multi-layer nanofibers due to their hydrophobic characteristics when they are organized in multi shell layers [100,101].

The restorative viability of anticancer medicinal nanofibers classification is expanded by (A) planned multi-drug/nanofibers preparation [102,103], (B) the preparation of nanofibers from such polymers that can sense both temperature and pH [55,67,86,104,105,106,107,108,109], (C) the fabrication of magnetic nanomaterials for the utilization of drug delivery and hyperthermia treatment [32,110]. The death rate due to cancer is increasing due to manufacturing/designing these carriers.

## 4. Commonly Used Polymers for Making Nanofibers for Cancer Treatment

**Figure 5:**
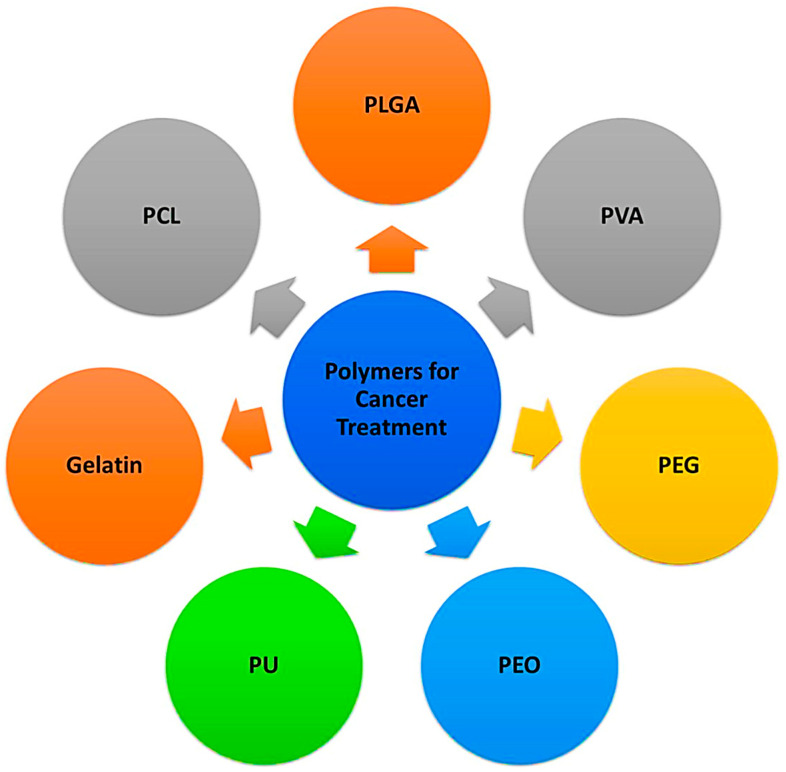
Commonly used polymers for nanofibers for cancer treatment.

Various kinds of polymers, as mentioned in Figure 5, have been accounted for in this review that can capture and convey cancer drugs to the particular area with continuous release. The decision of which drug, producing strategy, polymer, and post-modifications should be used are vital to consider when one needs to manage cancer-affected cells. Currently, the following polymers are being utilized in nanofibers-based disease treatment frameworks and systems.

### 4.1. PLGA (Poly(lactic-co-glycolic Acid))

Biodegradable and biocompatible, PLGA is a copolymer that is frequently employed in the manufacturing of nanofibers for the treatment of cancer. It is a well-known contender for medication delivery systems due to its capacity for controlled release. PLGA can be used in a variety of biomedical applications since it breaks down in the body into non-toxic metabolites [111].

### 4.2. PVA (Polyvinyl Alcohol)

PVA is a synthetic polymer that dissolves in water and is frequently used in electrospinning procedures to create nanofibers that are used in cancer therapy applications. Its advantageous qualities make it biocompatible and an efficient medication delivery system carrier [112].

### 4.3. PEG (Polyethylene Glycol)

Water-soluble and multipurpose, PEG is renowned for its superior biocompatibility. PEG improves the stability and biocompatibility of nanofibers and is frequently employed in conjunction with other polymers, which makes it appropriate for use in cancer treatment applications [113].

### 4.4. PU (Polyurethane)

PU is a biocompatible, flexible polymer that is used to make nanofibers for tissue engineering and medication delivery. Its application in the treatment of cancer is facilitated by its adaptability and drug compatibility [114].

### 4.5. Gelatin

Collagen is the natural source of the protein polymer known as gelatin. It is frequently mixed with other polymers to improve the mechanical characteristics and biocompatibility of nanofibers used in tissue engineering and cancer treatment [115].

### 4.6. PCL (Polycaprolactone)

PCL is a biodegradable polyester that is frequently utilized in cancer therapy nanofiber applications. PCL is appropriate for tissue engineering and sustained release medication delivery systems due to its slow rate of degradation [116].

### 4.7. Cellulose Acetate

In the presence of sulfuric acid, cellulose acetate can be achieved through the chemical reaction of cellulose with acetic anhydride and CH_3_COOH. A solution of cellulose acetate is needed for the manufacturing of fibers, which totally depends on degree of substitution in the cellulose acetate solution. A solution of cellulose acetate with the degree of substitution 2 or 2.5 is frequently soluble in different solution like dioxane, methyl acetate, and acetone and if the solution of cellulose acetate contains a degree of substitution greater than, 2.5 then it should be easily soluble in dichloromethane [117]. Cellulose and its mixes are often utilized to make nanofibers which need high absorbency [118]. Cellulose-based nanofibers have great advantages in the medical field for wound dressing and drug distribution owing to their enhanced characteristics like ecological and biological [119]. Suwantong et al. showed curcumin-loaded nanofiber mats made of cellulose acetate. When they placed these curcumin drug-based nanofibers on pig skin, these nanofibers displayed a slower discharge of curcumin. After checking the test result, it was found that 90 to 95% drug was discharged. Cellulose and its derivatives are valued for their exceptional absorbency, making them suitable for numerous applications. At the nanoscale, cellulose demonstrates remarkable mechanical properties, including a high tensile strength, stiffness, and flexibility. These attributes are particularly noteworthy as they persist across a wide range of moisture conditions, rendering cellulose nanomaterials highly versatile and desirable for developing advanced materials with tailored functionalities [120]. Cellulose acetate-based nanofibers displayed nontoxic nature against human dermal fibroblast [121].

### 4.8. Poly (Vinyl Alcohol)

Poly (viny alcohol) is utilized as a drug delivery agent and is known to be a biodegradable and biocompatible polymer [122]. The analysis of cancer in the initial phases is very crucial. Zhao et al. demonstrated that nanofibers which have diameters 460 nm like polyethyleneimine and PVA nanofibers which were controlled by hyaluronic acid can effectively analyze and capture cancer cells. The generated nanofibers were smooth and displayed an excellent efficiency [123]. Fan et al. used folic acid-based receptors to analyze the detection of cancer cells. They synthesized nanofibers like polyethyleneimine and poly vinyl alcohol and folic acid to check for the early stages of cancer [124]. Poly vinyl alcohol and core–sheath nanofibers loaded with DOX are utilized for the treatment of cancer cells in ovaries with a sustained discharge of drugs. These nanofibers were tested against SKOV3 disease cells and presented great outcomes [125].

### 4.9. Poly (Styrene)

Polystyrene is an engineered polymer and has an exceptionally low biodegradation rate. Iron oxide nanoparticles-based poly styrene nanofibers can be successfully utilized for the hyperthermic treatment of cancer cells [126]. Hyperthermia treatment is carried out to kill the dangerous cancer cells with the help of heat in a localized area. Iron oxide nanoparticles-based polystyrene nanofibers produce heat when they were uncovered to another magnetic field and destroy human ovarian cells completely in 10 min by becoming attached to their exterior part. Poly styrene nanofibers contain collagen on their surface which helps in capturing more cancer cells to these nanofibers. Against SKOV3 different results have been checked but the most considerable method for results verification was the alternating heating produced method. MDR Leukemia 562 cells have been treated with nanofibers (poly (N-isopropylacrylamide)-co PS) loaded with Daunorubicin; these nanofibers efficiently decreased the cells MDR and also increased the acceptance of this drug [127].

### 4.10. Poly (Hydroxyalkanoate)

Poly (hydroxyalkanoate) are green materials which can be formed by many microbes [128]. Because of their outstanding characteristics, these nanofibers are used in medical applications [129]. Poly (hydroxyalkanoate) has been shown to be acceptable for clinical purposes, and they are excellent for use in cell multiplication and tissue recovery without the danger of producing cancer cells [130]. The discharge rate of a drug is totally depended on the parameters of electrospinning like size and structure [131]. Due to the unevenness of the surface, poly (hydroxyalkanoate) nanofibers were found to display a reduced crystallinity in comparison with bulk film but to express an amplified angle of contact when checked against bulk film [132].

Cationic peptides have shown the greatest anticancer possibility but in the result of depolymerization of poly (hyroxyalkanoate)-conjugated R10 and R-3- hydroxy decanoic acid R10 are obtained with cationic peptides to increase the efficiency of peptides against cancer. Extra cytotoxic effects have been observed due to the conjugated peptides compared to unconjugated peptides in the contradiction of T cells leukemia, human pancreatic carcinoma, lung carcinoma, human glioma, and colorectal cancer [133]. Poly (hydroxyalkanoate) has been used as a nanocarrier for cellular treatment [134]. Five different forms of drugs have been incorporated into nanofibers (collagen peptides, Poly(3-hydroxybutyrate-co-3-hydroxyvalerate) used against gastric cancer cells [135]. Therefore, these nanofibers are mostly used in the medical field due to their green nature and ecofriendly behavior. But, the commercialization of these nanofibers is very difficult because these polymers are very costly due their preparation from microbes [136,137,138].

### 4.11. Peptides

Peptides are small chain monomers that are interconnected with amide bonds. Zhang et al. [139] made RADA16-I peptide electrospun nanofibers that exhibited excellent characteristics for three dissimilar categories of ovarian cells. The oral path is more challenging for the delivery of intact peptides because of the acidic pH in the digestive system (stomach). Arginine–glycine-aspartic acid relates to peptide nanofibers that can effectively transfer (curcumin) to the targeted area. Embryonic kidney, breast cancer cells and liver carcinoma cells are three diverse types of cancer cells which have been efficiently analyzed with the help of curcumin-based loaded peptides nanofibers [140]. L-Peptides and D-Peptides’ stability was checked by Yang et al. both in vitro and in vivo, and different behaviors were shown when they were used to vaccinate mice which was likely due to the different bio supply [141].

### 4.12. Chitosan

Chitosan can be prepared from many materials like nanobeads [142], sponges [143], membranes [144,145], microparticles [146], hydrogels [147], scaffolds [148], nanoparticles [149], and nanofibers [150]. Chitosan is a widely studied naturally occurring polymer broadly used for medical applications, medicine transfer, and tissue engineering [151]. Nanofibers of chitosan are mostly used in clinical applications [152,153]. Ardeshirzadeh et al. demonstrated the feasibility of DOX drug-based Chitosan, polyethylene oxide, and graphene oxide nanofibers for the efficient treatment of cancer. However, a π-π marking between DOX and graphene oxide caused a reduced medicine loading proficiency in Chitosan, polyethylene oxide, and in graphene oxide fibers than Chitosan/polyethylene oxide electro spun drug loaded filaments. These nanofibers displayed pH-dependent release of a drug, and when the pH was 7.4, constant discharge of drug was noticed. An in vitro cytotoxicity examination exhibited an improved resistance to the growth of A549 cells after 72 h in comparison with a free DOX drug which could be credited to the slow and maintained discharge of the medicine [154]. However, anti-cancer and other different characteristics of Chitosan have been demonstrated well at this point, but the important thing is the continuous discharge of drug for long time period as Chitosan is water loving and its expanding performance permits a simple diffusion of medication from filaments. It has additionally been realized that chitosan cannot be electrospun effectively without any secondary polymers and the mostly of them are water loving like poly vinyl alcohol and polyethylene oxide, etc. Therefore, the continuous discharge of a drug for long time period is still under investigation through post modifications or alterations to further polymers.

Their also some drugs used for the cancer treatments as mentioned in Table 1.

## 5. Methods for Producing Nanofibers

Nanofibers can be prepared by various methods such as force spinning [155], interfacial polymerization [156], phase separation [157], self-assembly [158], wet spinning [159], drawing [160], template melt extrusion [161], thermal induced phase separation [162], melt blowing [162], template synthesis [163], chemical vapors deposition [164], and electrospinning [165]. The multi-needle and multiple-jet needless spinnerets techniques of electrospinning are most commonly utilized in the research work and industrial services for the production of nano and microfibers at small and large scale [166,167,168].

In 1861–1903 Morton and Cooley developed the advanced electrohydrodynamic shower strategy to scatter liquids by electrostatic power [169]. Until 1990, this strategy did not achieve much consideration by experts. On the other hand, between 1934 and 1944 and furthermore between 1855 and 1944, there were a few authorizations on the electrospinning arrangement for the creation of polymeric strands [170,171,172,173].

### Production of Nanofibers through Electrospinning

**Figure 6:**
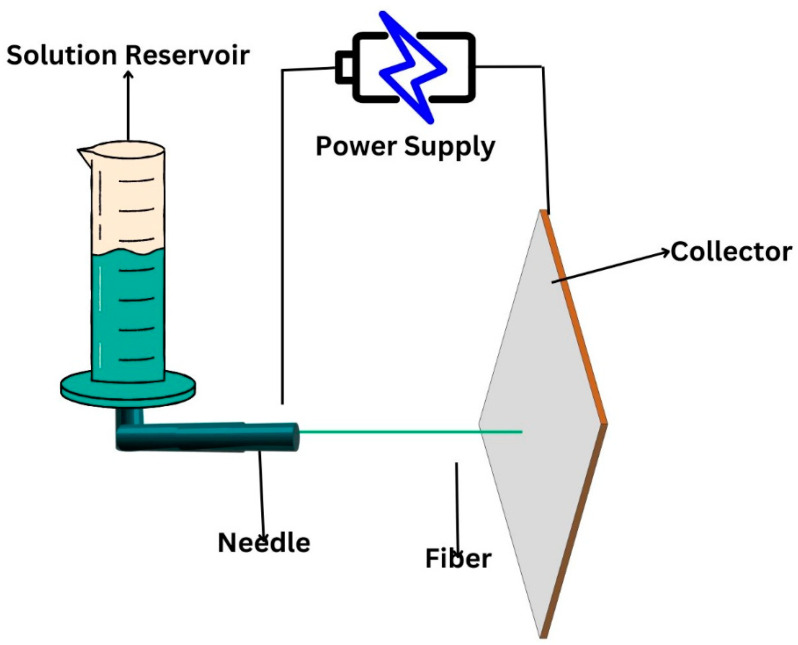
Production of nanofibers through electrospinning.

In the system used for electrospinning, polymer solutions are softened under high electric power and the supply is squeezed out from a jet and then it dries and is collected on drum as shown in Figure 6. Different parameters impact the electrospinning system, like the polymer solution and their characteristics, electrospinning machine characteristics, environment, and surroundings [174].

There are some parameters while discussing nanofiber production through electrospinning as mentioned in Table 2.

## 6. Approaches for Controlled Drug Release from Nanofibers

### 6.1. Control of Drug by Polymer

**Figure 7:**
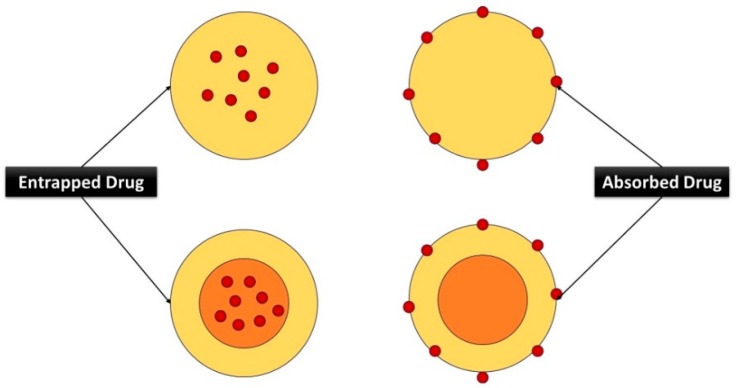
Control of drugs by polymers.

The polymers electrospun into nanomaterials have large effect on drug discharge and control. Different polymers show different discharge mechanisms for drugs. Hydrophilic drug-loaded nanofibers are the best polymers for acquiring a burst discharge effect. Hydrophilic nanofibers such as PVA have been allowed for the development of a quick dissolving drug conveyance nanofiber to transport caffeine and riboflavin drugs as mentioned in [175]. These nanofibers delivered the medications in a ruptured way because of the high water-loving nature of the polymer. Within 60 s, 100 percent of the caffeine and nearly 40 percent of the riboflavin was discharged [175]. Likewise, nanofibers of polyvinylpyrrolidone have shown an 84.9 percent ibuprofen discharge in just 20 s for a quick dissolving oral medication conveyance system [176]. Due to the nature of the fibers, PVP nanofibers loaded with Loratadine showed a high burst discharge [177]. A very strong burst effect has also been observed in the case of a burn wound when using nanofibers for a rapid pain-relieving action [178]. Water-repellent polymers can be utilized when a maintained discharge of medicines is expected from nanofibers. Hydrophobic co-polymers such as poly (lactic-co-glycolic acid) have exhibited sustained anticancer drug discharge for 60 days [179]. Nanofibers like PCL, poly lactic acid, and PVA express diverse phenomena in drug discharge because of the character/structure of the polymers [180]. Consequently, the selection of an appropriate polymer for favorable drug discharge is crucial. The mechanism of controlling release of drugs by polymers is illustrated in Figure 7.

### 6.2. Control of Drug by Structure

**Figure 8:**
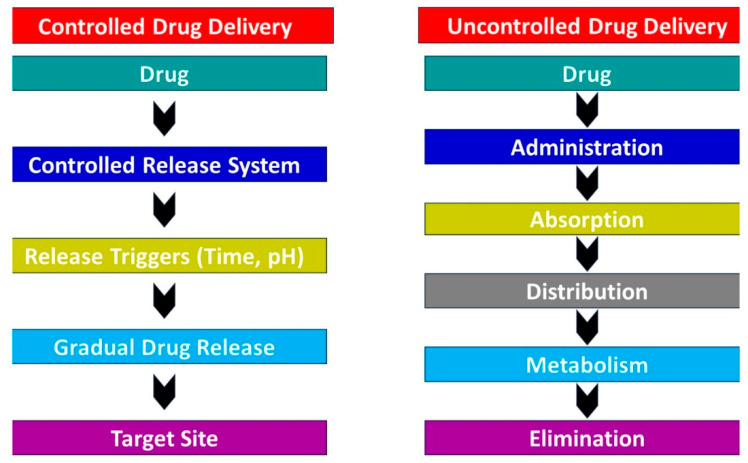
Controlled and uncontrolled drug delivery mechanism.

Changing the electrospinning parameters (drug loading quantity, mixing/blend, nanofibers layers, size of fibers and affinity of drug with polymers) also changes the structure of fibers. A small modification to the structure of nanofibers can alter the rate of medicine release. Two different structures of fibers loaded with the same amount of drug show two different releasing phenomenon because of a change in the dissemination path. For example, the rate drug release by flat nanofibers is higher than that of rounded nanofibers because there is less distance between the drug and the edges. Recently, round and flat nanofibers have been found to show both slow and fast releases of drugs when loaded with diclofenac sodium [181]. The pore size of nanofibers is another significant boundary to control the medicine discharge. A bigger pore size leads to burst discharge because of the ease of medication dissemination. Polyhydroxyalkanotes nanofibers filled with paclitaxel anticancer drug showed a quicker discharge when the pore size was bigger [131]. Nanofibers with a larger diameter show more diffusion of drugs in comparison to nanofibers with small diameters. Various diameters of nanofibers can be achieved by altering the settings of electrospinning. The speed of the discharge of a drug from nanofibers of poly l-lactic acid show faster and slower discharges from greater to smaller diameters [182]. The development of core–sheath nanofibers is another way to reduce the amount of a drug released by nanofibers. When maintaining all the other parameters (structure, pore size, porosity and diameters), polycaprolactone nanofibers presented less discharge of drugs in comparison to blended nanofibers. Core–sheath fibers showed a drug release of 34% while the blended nanofibers showed a 60% drug discharge in the same time frame [183]. Therefore, by changing the electrospinning conditions, drug release can be controlled as per the requirements by modifying the structure of nanofibers as mentioned above. The difference between controlled and uncontrolled drug delivery mechanism is illustrated in Figure 8.

### 6.3. Blend-Dependent Release

Mixing one or more polymers together can be used as a compelling tool to make different drug discharge profiles. In an investigation, just a 1.5% discharge of ciprofloxacin has been observed in 40 days by poly (methyl methacrylate) nanofibers. The drug-releasing phenomenon of ciprofloxacin was changed by blending some water-loving polymers like chitosan, PVA, and poly (ethylene oxide). The mixing of 10 percent chitosan with poly (methyl methacrylate) showed a constant discharge of ciprofloxacin, the mixing of PMMA with poly (ethylene oxide) exhibited burst discharge of ciprofloxacin, and mixing with PVA depicted a combination of both constant and burst discharge of ciprofloxacin [177]. The mixing of different polymers has been proven to be very useful in the phenomenon of drug discharge.

### 6.4. Drug-Dependent Release

The discharge of medicine from different nanofibers relies on the different drug characteristics. A hydrophobic polymer is more suitable for use with a hydrophobic drug while a hydrophilic drug is more suitable for use with a hydrophilic polymer. Several issues arise like the drug becoming attached to the outer layer of fibers which are enclosed and burst discharge due to mixing a hydrophilic polymer or drug with a hydrophobic polymer or drug [184,185].

The final discharge of medicines/drug highly depends on the quantity of drug loaded in the nanofibers. Nanofibers loaded with a large mount of a drug are needed for early burst discharge [186]. The expansion of molecular mass and collaboration among polymers and drug maintains the steady discharge of a drug [187]. To achieve both constant and burst discharge simultaneously, a combination of hydrophilic and hydrophobic drugs are useful; water-repellent drug will discharge slower and water-loving drugs will discharge quickly, and nanofibers composed of gelatin and PLGA nanofibers loaded with effective anticancer drugs (camptothecin and doxorubicin hydrochloride), which are the combination of both hydrophilic and hydrophobic anticancer drugs, have shown a high-level anticancer effect against HepG-2 cancer cells because of the constant discharge of doxorubicin hydrochloride and the burst discharge of camptothecin [188]. Thus, the hydrophobic and hydrophilic character of these drugs plays a vital role in the discharge of drug and also the polymer to which these drugs are attached.

### 6.5. Post Modification Release of Drug

The continuous discharge of medication can be accomplished by the post adjustment of the composed nanofibers. There is a possible solution to controlling the drug discharge. Chemical and plasma alterations to nanomaterial can initiate the medication discharge [189,190]. Different post-change methods have been used by analysts to alter the medication discharge from nanofibers, like interconnecting them with synthetic compounds or particles through heat action and ions. These techniques are helpful to give permanency to altered nanofibers against water which decreases the medication discharge. For instance, nanofibers of PVA adjusted using the interconnected method with methanol or post-heat treatment have been displayed to lessen the disintegration in water, thus physical firmness can be attained [191]. PVA nanofibers loaded with non-flammable drug dexapanthenol, link up with ammonium peroxydisulphate have been observed to encourage continuous discharge of drug [192]. Likewise, the burst discharge of PVA nanofibers loaded with ketoprofen was shown to decrease when they were linked with methanol [193]. In this manner, an appropriate assortment of post modification treatment can be utilized to alter the medicine discharge from nanofibers.

Controlled drug release from nanofibers is critical for optimizing therapeutic efficacy. Various strategies can be employed to modulate drug release kinetics, including polymer selection, fiber morphology, and drug loading. To achieve targeted drug delivery, stimuli-responsive nanofibers have emerged as a promising approach. By incorporating pH-sensitive or thermoresponsive polymers into the nanofiber matrix, drug release can be triggered by changes in the physiological environment. For instance, pH-sensitive nanofibers can be designed to release their therapeutic payload in the acidic tumor microenvironment, enhancing drug concentration at the target site. Similarly, thermoresponsive nanofibers can be formulated to respond to temperature variations within the body, enabling drug release in specific tissues or organs. These advanced strategies offer the potential to improve drug delivery efficiency, reduce systemic toxicity, and enhance therapeutic outcomes [194].

## 7. Future Directions 

**Figure 9:**
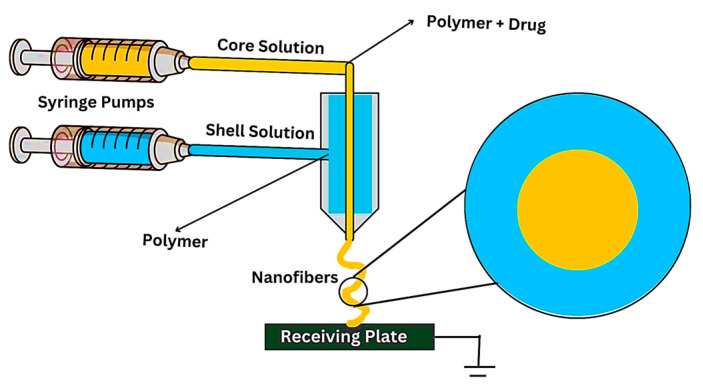
Electrospinning method to prepare nanofibers web for cancer treatment.

Electrospinning is a process used to create ultrafine fibers by applying a high-voltage electric field to a polymer solution or melt as illustrated in Figure 9. In coaxial electrospinning, two or more solutions are spun simultaneously through separate concentric spinnerets, resulting in core–shell or multilayered fibers shown below.

In coaxial electrospinning for cancer treatment, the core–shell structure allows for the encapsulation of therapeutic agents, such as chemotherapeutic drugs, proteins, or nanoparticles, within the core of the nanofibers. The shell, typically made of a biocompatible polymer, provides protection and allows for the controlled release of the therapeutic agent.

Coaxial electrospinning offers several potential advantages for cancer treatment, including an enhanced drug loading capacity, the improved stability and bioavailability of therapeutic agents, and the ability to tailor the properties of the nanofibers for specific applications.

Overall, coaxial electrospinning holds promise as a novel approach for the development of advanced drug delivery systems for cancer treatment, offering the potential for improved therapeutic outcomes and reduced side effects compared to conventional treatment modalities. However, further research and development is needed to optimize the design and fabrication of coaxial electrospun nanofibers for clinical applications.

## 8. Conclusions

The traditional method of conveying a drug to the affected area relies on the control and continuous drug discharge of the drug. The clinical sciences have been supported by utilizing nanotechnology-based frameworks. The designated and continuous drug discharge achieved with these nano-transporters shows that it is a promising methodology. Nanofibers show supported drug discharge compared to free drugs and, consequently, are more effective against cancer cells. The choice of the drug and polymer is vital in deciding on the right blend for supported drug conveyance. Nanofibers can carry a high quantity of various anticancer medications; naturally extracted materials are helpful treating cancer-affected cells and magnetic nanoparticles have also proved their worth in blends with other cancer-removing drugs. The conveyance of a hydrophilic and hydrophobic drug like curcumin can generally be controlled with the assistance of nanofibers. Therefore, nanofibers have mind blowing benefits in protecting humans against cancer diseases.

## Figures and Tables

**Table 1:** Commonly used drugs in cancer treatment.

Drug Name	Application(s)
Paclitaxel	Breast, ovarian, lung, and pancreatic cancers
Imatinib	Chronic myeloid leukemia (CML), gastrointestinal stromal tumors (GISTs)
Tamoxifen	Breast cancer (especially hormone receptor-positive)
Rituximab	Non-Hodgkin lymphoma, chronic lymphocytic leukemia (CLL)
Cisplatin	Various solid tumors, including testicular and ovarian cancers
Trastuzumab	HER2-positive breast cancer, gastric cancer
Methotrexate	Various cancers, including leukemia and lymphomas
Bevacizumab	Colorectal, lung, breast, and kidney cancers

**Table 2:** Parameters for nanofiber production through electrospinning.

Parameter	General Range	Role in Electrospinning
Temperature (°C)	20–30	Affects solvent evaporation rate and solution viscosity. Higher temperatures can dry fibers faster but may also lead to defects.
Viscosity (cP)	100–1000	Influences jet stability and fiber diameter. Higher viscosity generally leads to thicker fibers.
Flow rate (µL/min)	0.1–10	Controls the amount of solution pumped and affects fiber diameter and morphology.
Voltage (kV)	5–30	Determines the electrostatic force on the jet, influencing fiber stretching and diameter. Higher voltage generally leads to thinner fibers.
Needle collector distance (cm)	5–20	Affects solvent evaporation and fiber alignment. Shorter distance can lead to thicker fibers and slower drying.

## Data Availability

Not applicable.
